# Assessment of T Cell Receptor Complex Expression Kinetics in Natural Killer Cells

**DOI:** 10.3390/cimb44090265

**Published:** 2022-08-25

**Authors:** Khder H. Rasul, Alamdar Hussain, Hazel Reilly, Maria Karvouni, Carin I. M. Dahlberg, Mustafa S. Al-Attar, Arnika K. Wagner, Evren Alici, Dara K. Mohammad

**Affiliations:** 1 Center for Hematology and Regenerative Medicine (HERM), Department of Medicine Huddinge, Karolinska Institutet, SE-141 83 Stockholm, Sweden; 2 Department of Biology, College of Science, Salahaddin University-Erbil, Erbil 44002, Kurdistan Region, Iraq; 3 College of Agricultural Engineering Sciences, Salahaddin University-Erbil, Erbil 44002, Kurdistan Region, Iraq

**Keywords:** T cell receptor, natural killer cells, TCR positive natural killer cells, antigen, cancer cells

## Abstract

Among the polypeptides that comprise the T cell receptor (TCR), only CD3ζ is found in Natural Killer (NK) cells, where it transmits signals from activating receptors such as CD16 and NKp46. NK cells are potent immune cells that recognize target cells through germline-encoded activating and inhibitory receptors. Genetic engineering of NK cells enables tumor-specific antigen recognition and, thus, has a significant promise in adoptive cell therapy. Ectopic expression of engineered TCR components in T cells leads to mispairing with the endogenous components, making a knockout of the endogenous TCR necessary. To circumvent the mispairing of TCRs or the need for knockout technologies, TCR complex expression has been studied in NK cells. In the current study, we explored the cellular processing of the TCR complex in NK cells. We observed that in the absence of CD3 subunits, the TCR was not expressed on the surface of NK cells and vice versa. Moreover, a progressive increase in surface expression of TCR between day three and day seven was observed after transduction. Interestingly, the TCR complex expression in NK92 cells was enhanced with a proteasome inhibitor (bortezomib) but not a lysosomal inhibitor (chloroquine). Additionally, we observed that the TCR complex was functional in NK92 cells as measured by estimating CD107a as a degranulation marker, IFNγ cytokine production, and killing assays. NK92 cells strongly degranulated when CD3ε was engaged in the presence of TCR, but not when only CD3 was overexpressed. Therefore, our findings encourage further investigation to unravel the mechanisms that prevent the surface expression of the TCR complex.

## 1. Introduction

Adoptive cell transfer of genetically modified effector lymphocytes has revolutionized cancer immunotherapy and inspired a plethora of novel strategies currently under investigation [1]. Among them, the generation of immune cells expressing engineered TCRs has shown potential [2,3,4]. TCRs are composed of αβ subunits displaying immunoglobulin-like variable domains, and they are associated with the CD3 complex formed by the γ, δ, ε, and ζ subunits [5]. TCR gene therapy involves the adoptive transfer of antigen-specific effector cells manufactured by transferring ectopic TCRα and TCRβ genes against a particular tumor-associated antigen/MHC complex [6,7]. Engineered TCRs are used in the allogeneic and autologous adoptive T cell immunotherapy [8], where they mediate cancer regression in humans [9]. Although promising, producing adequate numbers of engineered patient or donor T cells faces logistic and financial challenges. In addition, mispairing endogenous and genetically transferred TCRαβ chains is another obstacle to the successful development of the TCR-based immunotherapy [10,11]. Due to these drawbacks, other candidate effector immune cells have been considered the basis of TCR-expressing cell therapy.

Like cytotoxic CD8^+^ T cells, NK cells can kill target cells through potent cytotoxic mechanisms [12]. NK cell activation is reflected by a change in the stimulation balance of germline-encoded receptors [13]; it is independent of major histocompatibility complex (MHC)-mediated antigen presentation [14]. Recent advances in genetic manipulation technologies/strategies have made genetic engineering of NK cells feasible and increased antigen-specific cytotoxic activity even when expressing chimeric antigen receptor (CAR) constructs based on T cell activating domains [15,16,17]. Utilizing NK cells for TCR gene therapy mitigates the risk of mispairing, as they lack TCRαβ chains and downstream signaling CD3 chains (CD3δ, CD3γ, and CD3ε) except CD3ζ. Recently, the combination of TCR and CD3 chains has been shown to induce TCR expression on the plasma membrane of NK92 cells [18,19] and on peripheral blood NK cells [20]. However, introducing TCR/CD3 components into NK92 cells and other sources of NK cells is still challenging from a manufacturing and cost-effective viewpoint. Moreover, the expression kinetics of the TCR complex in NK cells are currently understudied.

This study aims to investigate TCR processing in NK cells, as the expression and processing of this protein may not be the same as in T cells. Here, we focused on the elements that critically regulate the half-life and expression of a TCR complex in NK cells. Additionally, we aimed to improve the transgene design, manufacturing procedure, time, cost, and yield of TCR-expressing cells. Our study showed that TCR-associated polypeptides were essential for TCR expression on the plasma membrane of NK92 cells. We also demonstrated that maintaining transduced NK92 cells in culture for a longer period and adding bortezomib (proteasome inhibitor) increased the surface expression of the TCR complex. The functionality of the transduced NK92 cells was determined by degranulation and cytotoxicity assays. Lastly, NK92 cells expressing the TCR complex highly degranulated upon CD3ε stimulation, but not when only CD3 was overexpressed.

## 2. Materials and Methods

### 2.1. Cell Lines and Plasmids

Human embryonic kidney 293 FT (HEK293FT), Jurkat, Human B cells immortalized with Epstein Barr virus (EBV B), and NK92 cell lines were purchased from the American Type Culture Collection (ATCC). HEK293FT cells were cultured in Dulbecco’s Modified Eagle Medium (DMEM, GIBCO, Waltham, MA, USA) supplemented with 10% heat-inactivated Fetal Bovine Serum (HI FBS, GIBCO, Waltham, MA, USA). HEK293FT media was additionally supplemented with 1% l-Glutamine Solution (Sigma–Aldrich, St. Louis, MO, USA), 1% Sodium Pyruvate Solution (Sigma–Aldrich), 1% non-essential amino acid solution (Sigma–Aldrich), and 2% 4-(2-hydroxyethyl)-1-piperazineethanesulfonic acid (HEPES) solution (Sigma–Aldrich) during viral production. Jurkat and EBV B cells were grown in Roswell Park Memorial Institute medium 1640 (RPMI, GIBCO, Waltham, MA, USA) supplemented with 10% FBS. NK92 cells were maintained in a Stem Cell Growth Medium (SCGM, Cellgenix, Freiburg, Germany) medium supplemented with 20% HI FBS and 500 IU/mL of recombinant human interleukin-2 (rhIL-2, Proleukin, Novartis, Basel, Switzerland) was added every other day. Cells were split every 2–3 days to maintain optimal cell density and incubated at 37 °C in a humidified 5% CO2/95% air incubator. The TCR and CD3 plasmids were kindly provided by Zelluna Immunotherapy AS company (Oslo, Norway). The TCR expression construct was designed by fusing the TCR alpha and beta chains with a 2A self-cleaving site. The CD3 expression construct consists of all four CD3 invariant chains (CD3ζ, CD3δ, CD3ε, and CD3γ), linked via 2A sites, as described in Mensali (2019). The inserts were cloned into a pCCL expression vector and used to produce 3rd generation lentiviral particles [18].

### 2.2. Lentiviral Production and Transduction of NK Cells

A calcium chloride-based chemical transfection method (CAPHOS, Sigma–Aldrich, St. Louis, MO, USA) was used to produce lentiviral vectors encoding GFP, TCR, NY-ESO-1 1G4, and CD3 (S1). Briefly, packaging plasmids gag-pol (pMDLg/pREE, plasmid#12251, addgene), Rev (pRSV-Rev, plasmid #12253, addgene), and VSV-G (pCMV-VSV-G, plasmid #8454, addgene)) were transfected into HEK293FT cells, and a lentiviral vector containing supernatant was collected 48 h later. The supernatant was concentrated using a Lenti-X^TM^ concentrator (TaKaRa Bio, Shiga, Japan), according to the manufacturer’s instructions. After titration and calculating infectious titer, a multiplicity of infection (MOI) 4 was used in each of the two lentiviral transduction steps (sequential transduction) to generate genetically modified NK92 cells. Specifically, 0.25 × 10^6^ NK92 cells were transduced in a 24-well plate (Falcon Corning, Kenilworth, USA) in the presence of 8 μg/mL of protamine sulfate (Sigma–Aldrich) and 6 μM BX795 (Sigma–Aldrich) in a final volume of 1 mL. The plates were centrifuged at 1000× *g* for 1 h at 32 °C, then incubated at 37 °C, 5% CO_2_ for 5 h. At the end of the incubation, cells were centrifuged at 300× *g* for 5 min at room temperature, and NK92 cells were resuspended in 0.5 mL of fresh complete SCGM medium. The following day, the second transduction was performed by removing media from the well (equal to 50 μL + lentivirus volume), adding 50 μL transduction mix (protamine sulfate + BX795 in media) and the required volume of lentivirus (MOI 4), and perform the remaining steps as above. Transduction efficiency was assessed on day three after the final transduction step by flow cytometry and positive cells were expanded for future experiments.

### 2.3. Flow Cytometry

Flow cytometry was used to determine the expression of transgenes on the plasma membrane and in the cytoplasm of NK cells, using standard procedures. For surface staining, cells were washed with phosphate-buffered saline (PBS) and stained with a live/dead cell marker at 4 °C for 20 min. After a washing step, cells were stained with antibodies at 4 °C for 25 min in PBS+ 2% FBS (FACS buffer). Labeled cells were washed and fixed with 1% paraformaldehyde (PFA) at room temperature (RT) for 20 min and acquired on a CytoFLEX S (Beckman coulter Life Science, Brea, USA) machine. Intracellular staining was performed using the BD Cytofix/Cytoperm^TM^ kit (Cat No. BDB554714), according to standard procedures. Cells were stained with live/dead and surface staining, as previously described, then fixed and permeabilized in fixation/permeabilization solution at RT for 10 min. Cells were washed by perm/wash buffer and stained with antibodies at RT for 25 min in perm/wash buffer. The labeled cells were washed with perm/wash buffer, and flow cytometry data acquisition was performed after resuspending labeled cells in PBS.

The antibodies used for staining NK cells were: CD56 (NCAM16.2) and IFNγ (B27) from (BD Biosciences, San Jose, USA). CD3ε (UCHT1), CD107a (H4A3) and CD56 (HCD56) from BioLegend. TCR (CH92) and NY-ESO-1 (IMMU 222), from (Beckman Coulter, Brea, CA, USA). While live dead stains, including LIVE/DEAD^TM^ Fixable Aqua Dead Cell Stain Kit, LIVE/DEAD^TM^ Fixable Far Red Dead Cell Stain Kit, and LIVE/DEAD^TM^ Fixable Near-IR Dead Cell Stain Kit from (Thermo Fisher Scientific, Waltham, MA, USA) were used. In addition, CellTrace^TM^ Violet (CTV) Cell Proliferation Kit from (ThermoFisher Scientific, Waltham, MA, USA) was used to label NK92 cells when co-cultured with target cells.

### 2.4. Treatment of TCR-Transduced NK92 Cells with Inhibitors

Two weeks after transducing NK92 cells with the TCR complex, cells were incubated with 25 μM of the lysosomal inhibitor chloroquine or the proteasome inhibitor 7.5 nM bortezomib for 20 h. Cells were harvested and stained for flow cytometry to see the influence of inhibitors on the percentage of TCR complex^+^ NK92 cells.

### 2.5. Peptides and Target Cell Pulsing

Lyophilized peptides were dissolved in dimethyl sulfoxide (DMSO), aliquoted, and kept at −20 °C until use. In a 6-well plate, 0.75 × 10^6^ antigen-presenting EBV B target cells were pulsed/incubated with 2 μL DMSO, 5 μM New York esophageal squamous cell carcinoma 1 (NY-ESO-1) peptide 157–165 (SLLMWITQC, GenScript, Lot: U528PFF230-9/PE0213), or 5 μM Transforming growth factor-beta receptor II (TGFbRII) peptide 131–139 (RLSSCVPVA, GenScript, Lot: U4344EK130-3/PE2507) for 16 h in 2 mL Opti-MEM media.

### 2.6. In Vitro NK Cell Activation

The peptide-pulsed EBV B cells were counted, washed, and co-cultured with sorted GFP^+^CD3^+^ and TCR^+^CD3^+^ NK92 cells for 4 h at an E:T ratio of 1:1 in the presence of CD107a PE-Cy7 clone H4A3 antibody throughout the assay in a final volume of 200 μL in U-bottom 96-well plates at 37 °C and 5% CO_2_. In another setup, genetically modified NK92 cells were also stimulated with plate-bound α-CD3ε clone OKT3 (5 or 10 µg). After the first hour of co-culturing, 0.7μL of Golgistop (BD Biosciences) was added to each well to block the intracellular protein transport process. In this degranulation assay setup, GFP^+^CD3^+^ NK92 cells were included as a control in all co-culturing conditions. The medium was used as a negative control, and phorbol12-myristate 13-acetate (PMA, 500 ng/mL, Sigma-Aldrich, St. Louis, MO, USA) plus ionomycin (500 ng/mL, Sigma-Aldrich) was used as a positive control. After the co-culture, cells were stained for surface (live/dead and CD56) and intracellular markers (IFNγ) as previously described in the flow cytometry section.

### 2.7. Labeling of Genetically Modified NK92 Cells

To discriminate effector cells from target cells, effector cells were labeled with CTV, as per the manufacturer’s instructions, prior to the co-culture assays. For live-cell IncuCyte imaging cytotoxicity assay, effector cells were labeled with CytoLight rapid green (Sartorius, Göttingen, Germany), as per the manufacturer’s instructions.

### 2.8. Flow Cytometry-Based and Live-Cell Imaging of NK92 Cell Cytotoxicity

Both flow cytometry and live-cell imaging were used to determine the cytotoxic capacity of sorted TCR-expressing NK92 cells. For the flow cytometry-based cytotoxicity approach, GFP^+^CD3^+^ and TCR^+^CD3^+^ NK92 cells were labeled with CTV to discriminate from the target cells during the analysis of acquired data. Labeled effector cells were co-cultured with pulsed target cells in a 1:3 ratio for 2 h, and whole-cell populations were collected and stained for flow cytometry.

An IncuCyte^®^ S3 system (Sartorius) was used for the live cell cytotoxicity assays, in which the IncuCyte machine was placed in a humidified incubator at 37 °C and 5% CO_2_. Effector NK92 cells, including GFP^+^CD3^+^ and TCR^+^CD3^+^ NK92 cells, were labeled by CytoLight Rapid Green Reagent. CytotoxRED (Sartorius) Working Solution (500 nM) was added to a final concentration of 250 nM to stain dead cells. Cells were co-cultured in a 1:1 E:T ratio in 96-well flat-bottom plate with 200 μL total volume. The plate was imaged (phase + green + red, four images) every 30 min for 9 h, then four images every 1 h for the rest of the assay.

### 2.9. Statistical Analysis

Flow cytometry obtained data was analyzed using FlowJo^TM^ 10 software (version 10.8.1). IncuCyte data was analyzed using IncuCyte software (v2019B). GraphPad Prism version 9.0. for Mac OSX (GraphPad Software, La Jolla, CA, USA; www.graphpad.com (accessed on 7 December 2021)) was used to generate graphs and perform statistical analysis (two-way ANOVA with Tukey’s multiple comparisons test).

## 3. Results

### 3.1. Influence of Time on TCR Complex Expression

**Figure 1:**
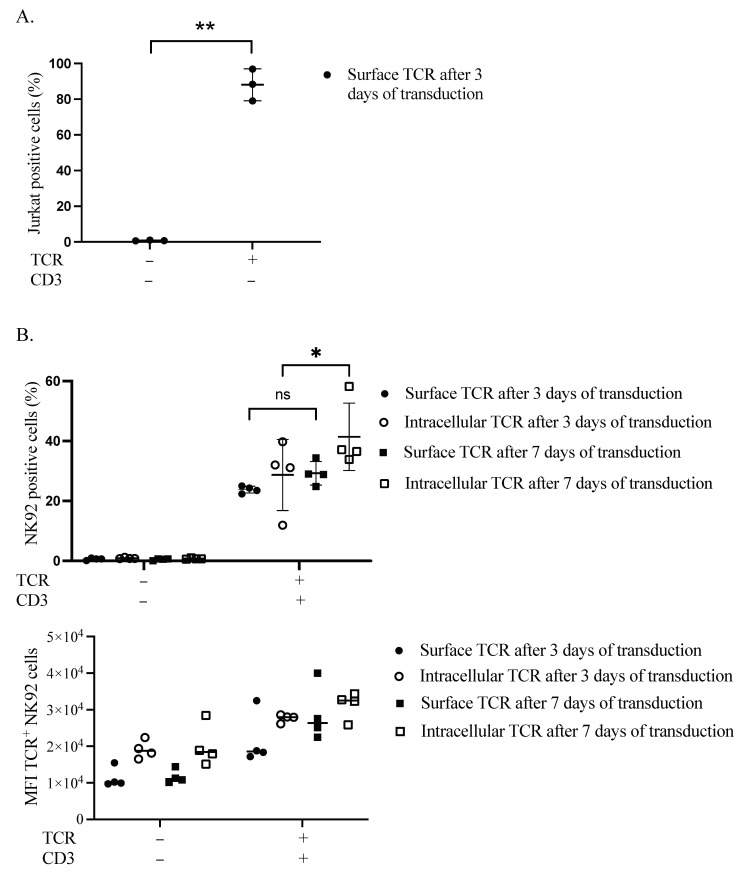
Retentions and dynamics of TCR expression in Jurkat and NK92 cells. (**A**) Confirmation of TCR expression in Jurkat cells. Three days after transduction, cells were harvested and stained for flow cytometry to assess the expression of TCR on the surface of Jurkat cells. Live cells were pre-gated during the analysis of acquired data. Three independent experiments were performed. The ** indicates a statistically significant *p* < 0.01. (**B**) Dynamics of transgene expression in NK92 cells. NK92 cells were transduced with TCR and then the day after they were transduced with CD3 subunits lentiviral vector. Three- and seven-days post-transduction, cells were harvested and stained for flow cytometry to see the expression of TCR. Live cells were pre-gated during the analysis of acquired data. Four experiments with sequential transduction and single transduction were carried out. ns indicates not significant. * *p* < 0.05.

We first verified the expression of our TCR transgene in the plasma membrane of Jurkat cells. After 3 days, more than 88% of cells were positive for TCR (Figure 1A and Figure S2). Secondly, we performed sequential transduction in NK92 to introduce the TCR chains, followed by the CD3 subunits. The transduction efficacy was estimated at two different time points, three and seven days after the CD3 subunits’ transduction. Surface expression of TCR in the plasma membrane of NK92 cells was delayed, as assessed by flow cytometry (S3). On day three, TCR surface and intracellular expression levels were 24.3% and 39.8%, respectively. Whereas on day seven, the surface and intracellular TCR expression levels were raised to 34.4% and 58.2%, respectively (Figure 1B).

### 3.2. TCR Complex Expression Requirements in the Plasma Membrane of NK92 Cells

**Figure 2:**
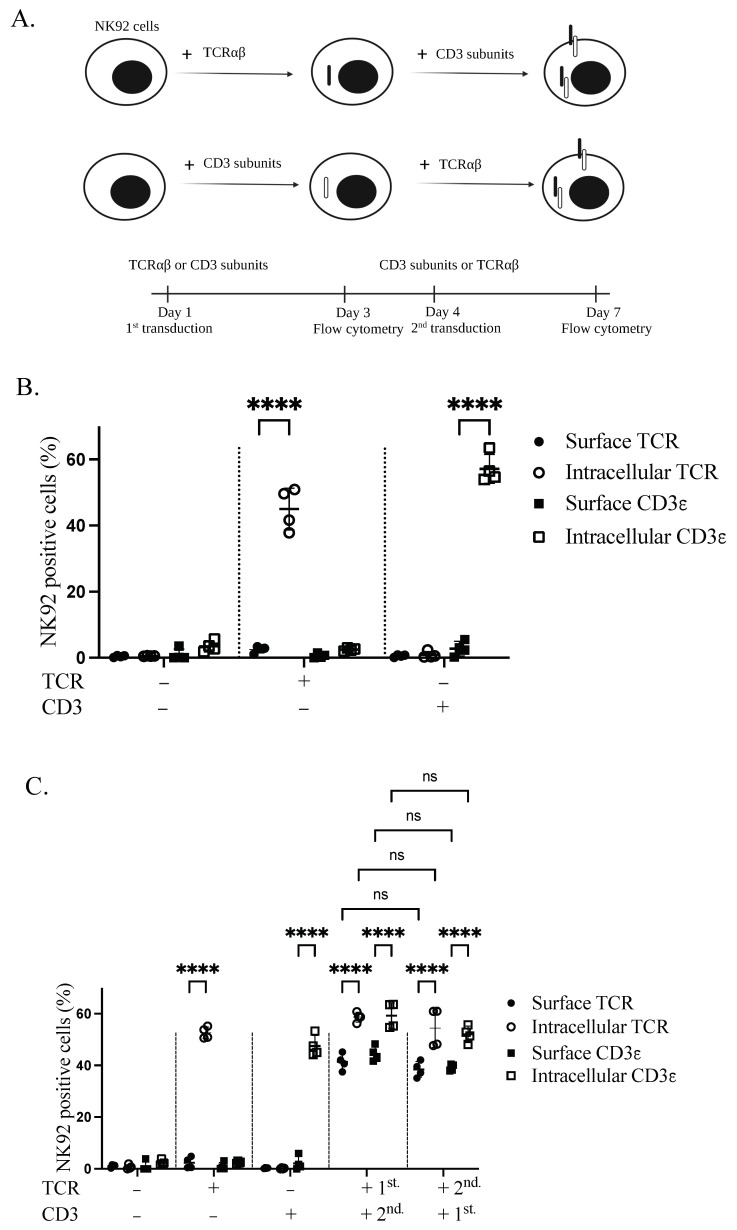
Transgenic TCR components are necessary to enable TCR complex expression on the cell membrane of NK92 cells. (**A**) Schematic overview of the sequential transductions and timeline. (**B**) Expression of TCR and CD3 alone in transduced NK92 cells. NK92 cells were transduced with either alpha/beta chains of TCR or CD3 subunits lentiviral vectors. Cells were collected and stained for flow cytometry three days after transduction. All cells were stained with TCR and CD3ε antibodies and live cells were pre-gated. Two independent experiments were performed. (**C**) NK92 cells were transduced with either TCRαβ or CD3 subunits lentiviral vectors. On day four of transduction, whole TCR and CD3 transduced NK92 cell populations were transduced with CD3 subunits and TCR lentiviral vectors, respectively. Cells were harvested and stained for flow cytometry seven days after first transduction to see the expression of transgenes. All cells were stained with TCRβ and CD3ε antibodies and live cells were pre-gated. Two independent experiments were performed. ns indicates not significant. **** *p* < 0.0001.

Stable expression of TCR complex in NK92 cells was achieved through lentiviral transduction. Sequential transduction was applied to produce genetically modified GFP^+^CD3^+^ and TCR^+^CD3^+^ NK92 cells. To test whether both TCRαβ and CD3 are necessary for TCR complex expression on the plasma membrane of NK92 cells, NK92 cells were transduced with either TCRαβ or CD3 subunits, according to the schematic representation (Figure 2A). Transduction with only one of the constructs revealed intracellular but not surface expression for both TCRβ and CD3ε transgenes (Figure 2B). After transducing the cells with the complementary second transgene (CD3 or TCRαβ), we identified the expression of both first transgenes on the cell surface. Specifically, 41.35% and 40.3% of cells were positive for TCRβ and CD3ε, respectively. However, the first transgene is still sequestered inside the cell in the absence of the second transgene. Interestingly, TCR or CD3 as the first transgene had no significant effect on the expression of the second transgenes in the plasma membrane of NK92 cells (Figure 2C).

### 3.3. Influence of Inhibitors on TCR Complex Expression

**Figure 3:**
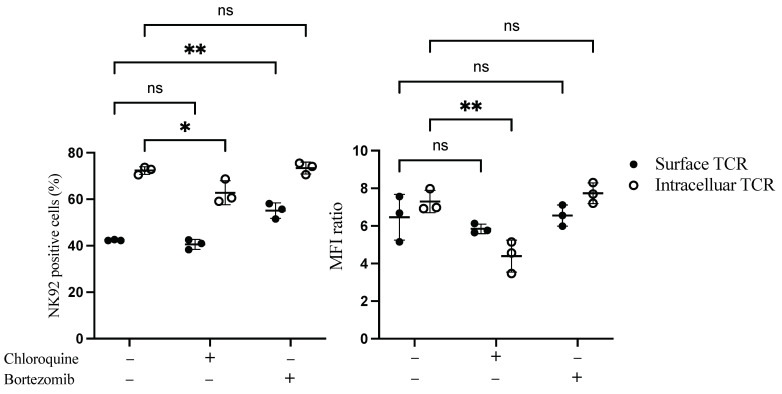
Flow cytometry-based TCR expression upon treatment of TCR/CD3 transduced NK92 cells with bortezomib and chloroquine inhibitors. NK92 cells were transduced with TCR as a first transgene on day 0, then transduced with CD3 subunits as a second transgene on day 1. Transduced NK92 cells were kept in the culture for at least for 2 weeks. Then they were incubated with proteasome inhibitor bortezomib or lysosomal inhibitor chloroquine for 20 h. Cells were harvested and stained for TCR expression assessment. Live cells were pre-gated during data analysis by FlowJo to obtain TCR-positive NK92 cells. Each dot represents the mean of duplicate data; three independent experiments were performed. ns indicates not significant. * *p* < 0.05; ** *p* < 0.01.

We next sought to examine whether the low surface expression of TCR on NK92 cells was due to degradation in the cytoplasm. Proteosome and lysosomal enzymes found in the cytoplasm of the cells are responsible for degrading misfolded, unfolded, and internalized proteins. Therefore bortezomib, a proteasome inhibitor, and chloroquine, a lysosomal inhibitor, were added to the transduced NK92 cells to assess their influence on TCR expression. Interestingly, bortezomib treatment significantly increased the percentage of surface TCR^+^ NK92 cells by 13.4%. In contrast, chloroquine significantly decreased intracellular transgene expression by 10% (Figure 3 and Figure S4).

### 3.4. Functional Evaluation of Genetically Modified NK92 Cells

**Figure 4:**
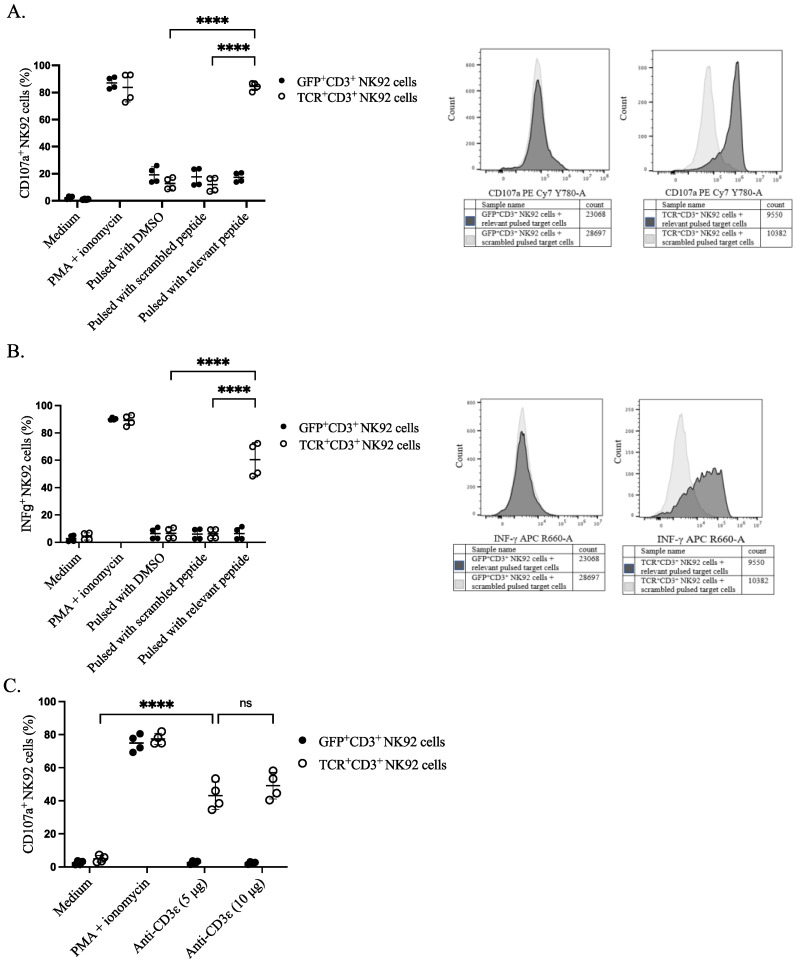
Functional properties of NK92 cells. NK92 cells were incubated either with prior pulsed target cells at an E:T ratio of 1:1 or with anti-CD3ε for 4 h. Golgistop was added after the first-hour incubation. CD107a conjugated PE-Cy7 antibody was present throughout the assay. Cells and supernatant were harvested for surface and intracellular staining on the same cells for flow cytometry. NK92 cells were separated from target cells by gating on CD56^+^ cells. (**A**) Percentage of CD107a positive NK92 cells after incubating with target cells, two independent experiments were performed. The **** indicates a statistically significant *p* < 0.0001. (**B**) Percentage of IFNγ positive NK92 cells after incubating with target cells, two independent experiments were performed. The **** indicates a statistically significant *p* < 0.0001. (**C**) Comparative assessment of transgene responses upon CD3ε agonistic treatment. The **** indicates a statistically significant *p* < 0.0001, and ns indicates not significant.

To assess the functional activity of genetically modified NK92 cells, we sorted the cells based on either TCR or GFP expression. The sorted cells were stimulated with peptide-pulsed target cells and their responsiveness was assessed by the CD107a surface expression as a degranulation marker and the production of the pro-inflammatory cytokine IFNγ (S5). Sorted TCR^+^CD3^+^ NK92 cells showed higher degranulation upon co-culture with relevant peptide-pulsed EBV B cells in comparison with all four control conditions: including negative control, co-culturing of GFP^+^CD3^+^ NK92 cells with pulsed target cells, effector NK92 cells with DMSO pulsed target cells, and genetically modified NK92 cells with scrambled peptide-pulsed EBV B cells (Figure 4A and Figure S6). A higher percentage of IFNγ was noticed in the effector gene-modified TCR^+^CD3^+^ NK92 cells upon co-culturing with relevant peptide-pulsed target cells than in all the control conditions mentioned above (Figure 4B and Figure S7). Similarly, anti-CD3ε (OKT3) stimulation (5 µg or 10 µg) also induced degranulation of NK92 cells only when the TCR complex was expressed (Figure 4C).

### 3.5. Specific Cytotoxicity of TCR^+^ NK92 Cells against Selective Target Cells

**Figure 5:**
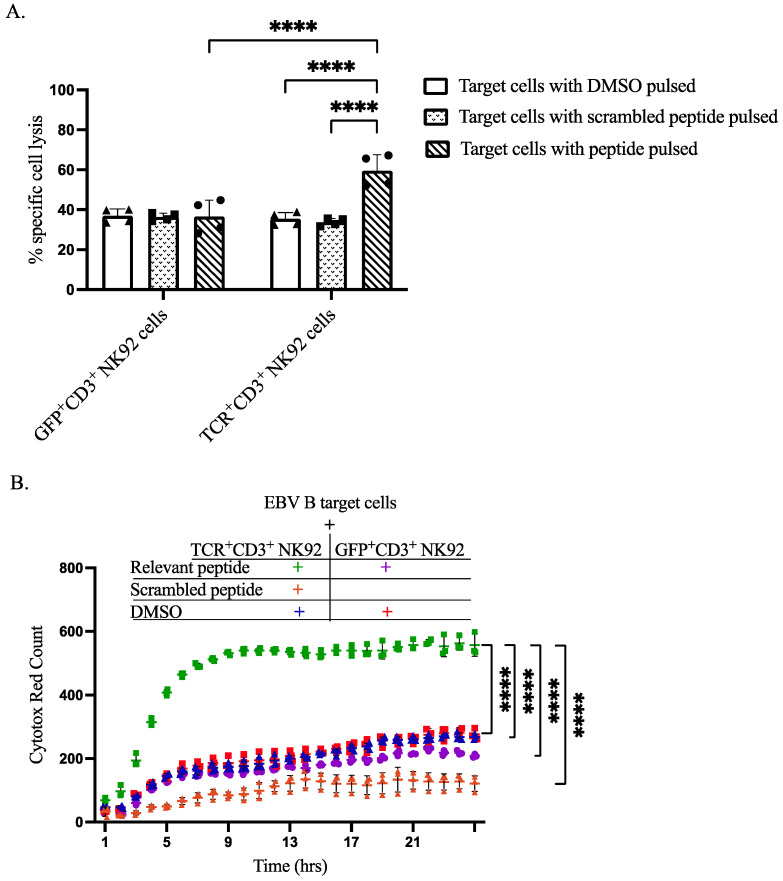
Cytotoxicity of sorted genetically modified NK92 cells upon co-cultured with target cells. (**A**) Flow cytometry-based In Vitro killing assay. Target cells were pulsed by incubation with DMSO, relevant peptide, or scrambled peptide for 2 h. Then genetically modified NK92 cells (GFP^+^CD3^+^ or TCR^+^CD3^+^ NK92 cells) were labeled with CTV and co-cultured with pulsed target cells at a ratio of 1:3 for 2 h. After incubation, whole-cell populations were harvested and stained for flow cytometry determinations. Flow cytometry data were analyzed by FlowJo, dead cells were gated from CTV negative cells. The **** indicates a statistically significant *p* < 0.0001. (**B**) In vitro cytotoxic live cell imagining activity of sorted genetically modified NK92 cells. To assess the cytotoxic activity of generated GFP^+^CD3^+^ and TCR^+^CD3^+^ NK92 cells by IncuCyte S3 live-cell analysis system, labeled NK92 cells were co-cultured with target cells at an E:T ratio of 1:1 without having peptide throughout the assay. The magnification power was the same for all images. Data analysis was performed from real live-cell images, and cytotox red counts as dead target cell markers were extracted per time. Normal death baseline was subtracted from each separately. Each dot represents the mean of triplicate data (each datum derived from four images). The **** indicates a statistically significant *p* < 0.0001.

To demonstrate the capacity of our genetically modified NK92 cells to recognize specific antigens and induce lysis of antigen-presenting target cells, we performed both flow cytometry-based assays and IncuCyte live-cell imaging analyses. CTV was utilized to differentiate between effector NK92 cells and EBVB target cells, as shown in gating strategy dot plots. Flow cytometry-based cytotoxicity assay revealed that co-culturing of TCR^+^CD3^+^ NK92 cells selectively killed target EBV B cells pulsed with relevant peptides at a level higher than all three control conditions (Figure 5A, Figures S8 and S9). IncuCyte live-cell imaging data further corroborated the selective killing of relevant peptide-pulsed target EBV B cells by TCR^+^CD3^+^ NK92 cells compared to control conditions (Figure 5B, Figures S10 and S11). Live cell imaging showed that co-culturing GFP^+^CD3^+^ NK92 with either pulsed DMSO or relevant peptide resulted in approximately the same level of killing target cells (Figure 5B, Figures S10 and S11). An increased level of target cell killing was noted after co-culturing EBV B target cells with TCR^+^CD3^+^ NK92 (Figure 5B and Figure S11).

## 4. Discussion

The current research aimed to explore TCR processing in NK cells, improve transgene design, increase the yield of TCR-positive NK cells, and decrease both the time and costs associated with generating genetically modified cells. TCR complex consists of an antigen-binding site (mainly TCRα and β chains, although there is a small population of T cells with γ and δ chains) and downstream signaling subunits (CD3δ, CD3γ, CD3ε, and CD3ζ chains). Since NK cells only express CD3ζ, we were interested in understanding whether TCR complex elements are necessary for the translocation of TCR into the plasma membrane of NK cells and the kinetics of cellular processing of the TCR complex in NK cells. In parallel, we generated MHC peptide-specific NK cells by inserting antigen-binding sites of TCRs in the presence of CD3 signaling subunits.

The present study showed that TCRαβ polypeptides and CD3 chains need each other to translocate or remain stable in the plasma membrane of NK92 cells (S12). TCRαβ or CD3, when expressed alone, were detected only in the cytoplasm but not on the cell surface. When both TCRαβ and CD3 were expressed, TCR was detected on the cell surface of NK92. Therefore, the synthesis and assembly of TCR components inside the cell are necessary for transport to the plasma membrane in NK92 cells, which is likely to be the same for other NK cell sources. TCR elements are expressed on the cell surface in a coordinated fashion after assembly in the endoplasmic reticulum. As shown in T cells, the assembly of the TCR multichain receptor complex is highly regulated as only correctly assembled receptors can reach the cell surface [21,22].

In our lab, the expression of various ectopically introduced TCRs was confirmed in transduced NK92 cells, suggesting that the plasma membrane of NK92 cells can support TCRs targeting different tumors. In general, the purpose of generating these genetically modified NK92 cells is to take advantage of both NK and T cell characteristics. NK cells expressing TCR might be reduced through specific antigen binding and innate responses upon loss/downregulation of the antigen. As for T cells, loss of target cells antigenicity is possible due to the MHC class I loss/downregulation [23,24,25]. Tumors can acquire specific MHC class allele loss after immune pressure, as seen in patients who relapsed following MHC-haploidentical stem cell transplant [26]. Furthermore, many tumors show down-regulation of HLA alleles [27]. Therefore, the dual cytotoxic function of our engineered NK92 cells provides an advantage over either NK or T-cell-based therapies.

Synthesis of TCRαβ polypeptides and the percentage of TCR^+^CD3^+^ NK92 cells were evaluated during a longer-term culture of the transduced cells. The TCR complex is a large multi-component complex consisting of multiple polypeptide chains that typically need a longer time to synthesize, assemble and translocate into the plasma membrane of non-T cells. The disproportionate presence of the various TCR complex members may lead to their degradation/recycling. Indeed, it has been shown in T cells that unassembled subunits or partial complexes do not progress to the Golgi apparatus, but rather are transported from the endoplasmic reticulum to the cytoplasm, where they are degraded [28]. Bortezomib is a proteasome inhibitor that might decrease this protein recycling or degradation since degradation of some of the subunits like TCRα takes place in the proteasome [29]. In contrast, partial CD3 complexes degrade in lysosomes of T lymphocytes [30]. The previous reports on T cells support our data showing an increased number of TCR^+^CD3^+^ NK92 cells after treatment with bortezomib. Collectively, these strategies can be considered a starting point in overcoming the many challenges that remain when generating TCR-positive primary NK cells.

Allogeneic NK cells do not cause graft versus host disease, making them a safer alternative to T cells [31,32]. In addition, NK cells lack TCR components except for CD3ζ, therefore can be used as a replacement for T cells by mitigating mispairing endogenous and genetically transferred TCRαβ chains [33,34]. NK cell lines [18,19] and peripheral blood NK cells [20] are transduced with TCR and can express the complex on their cell surface. Together, the NK cells mimicking the T cell phenotypes make an attractive candidate for an off-the-shelf TCR-based NK cell adoptive cell therapy. Therefore, the generation of different TCR complex^+^ NK92 cells is essential for creating a TCR NK cell biobank, for TCR-based cancer immunotherapy. We manufactured surface TCR^+^NK92 cells without cell sorting between two transductions, in the absence of having reporter gene in the constructs without using feeder cells as previous studies have used [20].

Regarding the activity of our manufactured cells, the percentage of CD107a degranulation marker on the surface of NK92 cells increased upon co-culture of TCR^+^CD3^+^ NK92 cells with EBV B target cells pulsed with relevant peptides. Similarly, stimulating CD3^+^ NK92 cells with plate-bound anti-CD3ε caused higher degranulation. In addition, IFNγ cytokine production as a marker of the functional activity of our generated NK92 cells was considerably higher upon co-incubation of the genetically modified TCR^+^CD3^+^ NK92 cells with relevant peptide-pulsed EBV B target cells. Results of other studies align with our data showing in terms of TCR^+^ NK cell functionality and in vitro response [18,19].

In addition, we wanted to assess the cytotoxicity of our genetically modified cells. TCR^+^CD3^+^ NK92 cells could selectively recognize antigens processed and presented by MHC class I on EBV B target cells. Moreover, according to the flow cytometry-based cytotoxicity and live cells imaging system, genetically modified TCR^+^CD3^+^ NK92 cells efficiently eliminated target cells pulsed with a relevant peptide. The gene expression profiling of TCR-NK92 emphasized the potential of these cells to acquire a phenotype for becoming a potent T-cell-like killer cell therapy product. The NK92 cell line is FDA-approved and highly cytotoxic against a broad range of tumor cells [35,36], demonstrating promising antitumor effects and clinical benefits without significant treatment-related side effects [37,38]. NK cells expressing TCR displayed MHC I restricted antigen detection and antigen-specific lysis of tumor cells [18,19,20], which is aligned with our results (Figure 5).

In conclusion, this study provided evidence that TCR elements can be separately expressed in the cytoplasm of NK92 cells but not on the plasma membrane. It requires the presence of the whole complex together to be stably expressed on the cell membrane, while the individual components can be found separately in the cytoplasm of NK cells (Figure 2C). We could also scale up the transduction efficiency by two different strategies, including keeping transduced cells in culture for a longer period and using a suitable titrated dose of bortezomib. We believe that these strategies will be helpful for the expression of big and multi-polypeptide chains. In addition, designing different antigens targeting TCR constructs and feasible expression of functional TCR complex proteins in the plasma membrane of NK92 cells provided the basis for creating a tumor-and peptide-specific TCR library. Altogether, the present study demonstrated that TCR-mediated NK responses could enhance NK cell efficacy against malignancies, especially when resistant to NK-mediated attack. Furthermore, TCR-positive NK92 cells can also compensate for MHC class I loss and associated immune-evasion strategy of TCR-mediated approaches, making TCR-positive NK92 cells unique in cellular therapy and adoptive cell therapy development.

## Data Availability

Not applicable.

## Supplementary Materials

The following supporting information can be downloaded at: https://www.mdpi.com/article/10.3390/cimb44090265/s1.
