# Supplementary material for: Assessment of T Cell Receptor Complex Expression Kinetics in Natural Killer Cells
Source: Curr Issues Mol Biol. 2022 Aug 25;44(9):3859–71. doi: 10.3390/cimb44090265 (PMC9497757; doi:10.3390/cimb44090265)
Supplement: Supplementary file 1 [file cimb-44-00265-s001.zip › cimb-1797642-supplementary.pdf]

Supplementary figures

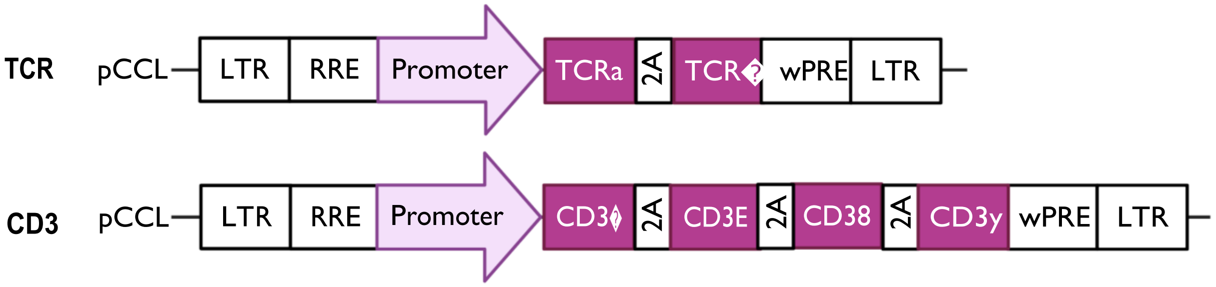

Supplementary figure S1: TCR and CD3 subunits backbones. Lentiviral vectors coding for TCRα/β and CD3-subunits.

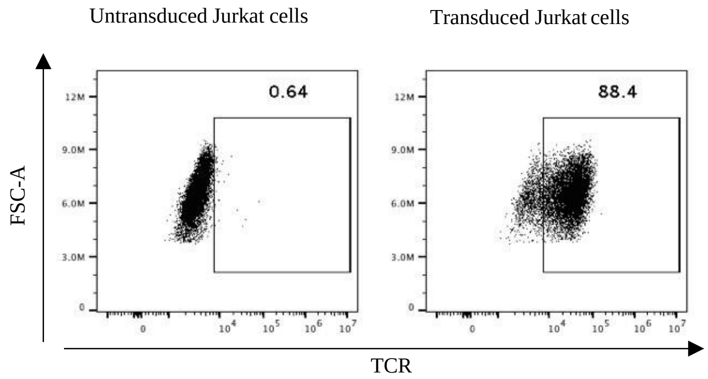

Supplementary figure S2: Surface-derived flow cytometry dot plot images display TCR expression in the plasma membrane of Jurkat cells after three days of transduction. All cells were stained with TCR antibody for flow cytometry data acquiring and gated on live cells during analysis by flowJo software.

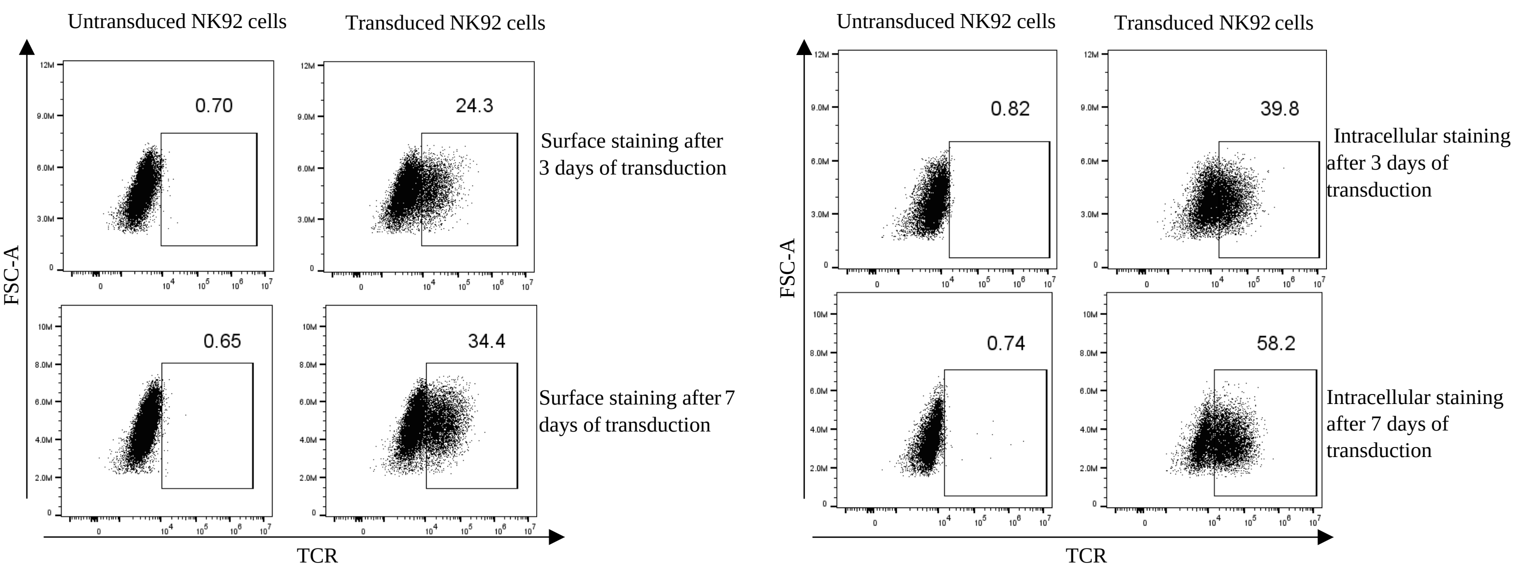

Supplementary figure S3: Surface and intracellular derived flow cytometry dot plot images displaying TCR expression in the plasma membrane of NK92 cells after three and seven days of transduction. All cells were stained with TCR antibody for flow cytometry data acquiring and gated on live cells during analysis by flowJo software.

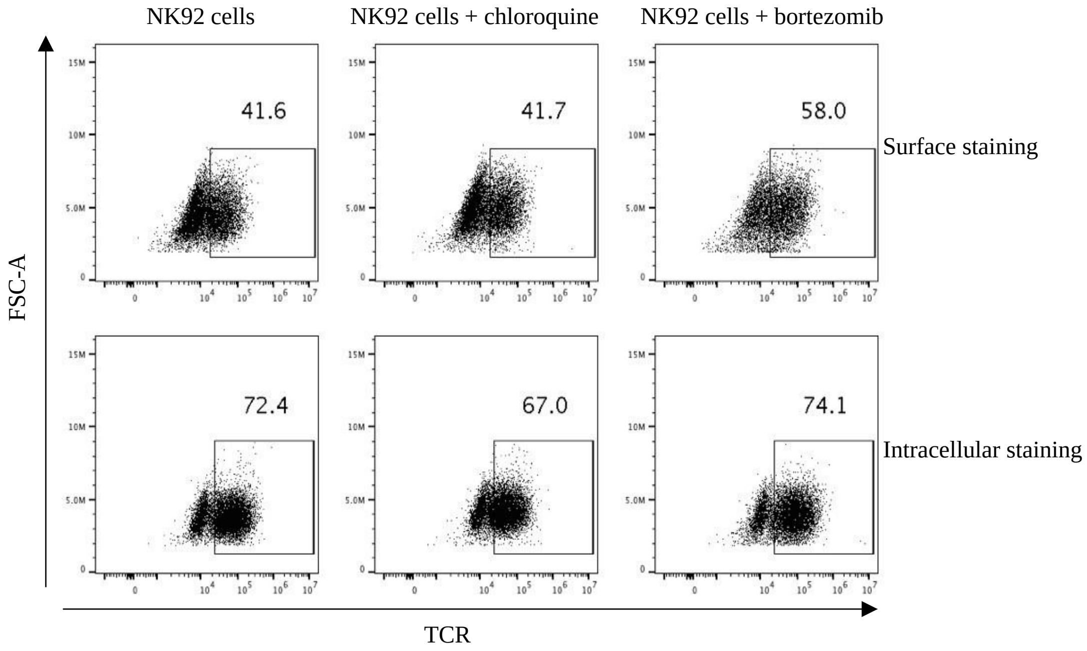

Supplementary figure S4: Surface and intracellular derived flow cytometry dot plot images present TCR expression in the plasma membrane of transduced NK92 cells after incubating with bortezomib and chloroquine inhibitors. All cells were stained with TCR antibody for flow cytometry data acquiring and gated on live cells during analysis by flowJo software.

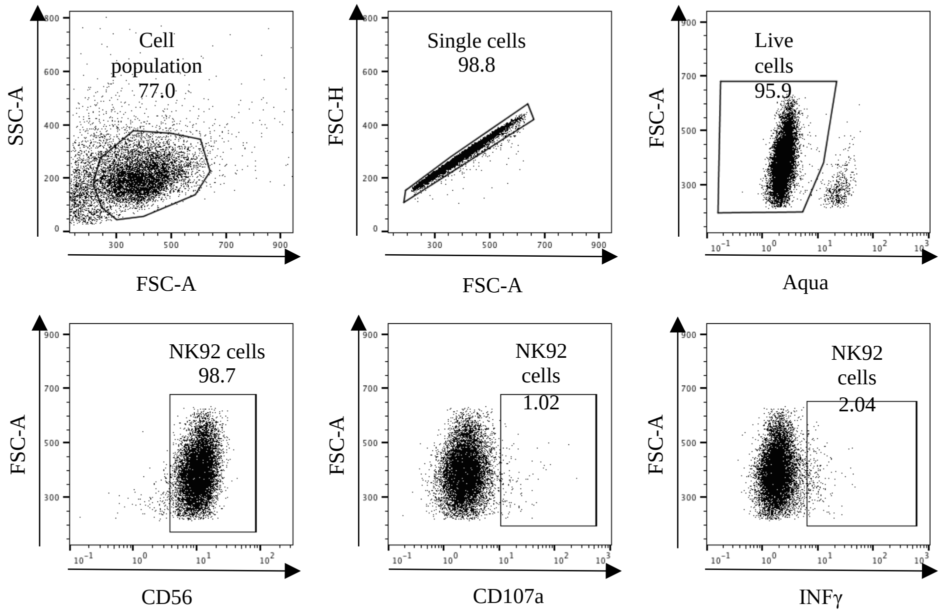

Supplementary figure S5: Flow cytometry dot plot images of self-medium stimulated negative control show the gating strategy, CD107a and IFNγ plots were gated on CD56 positive cells, and CD56 positive cells were live cells.

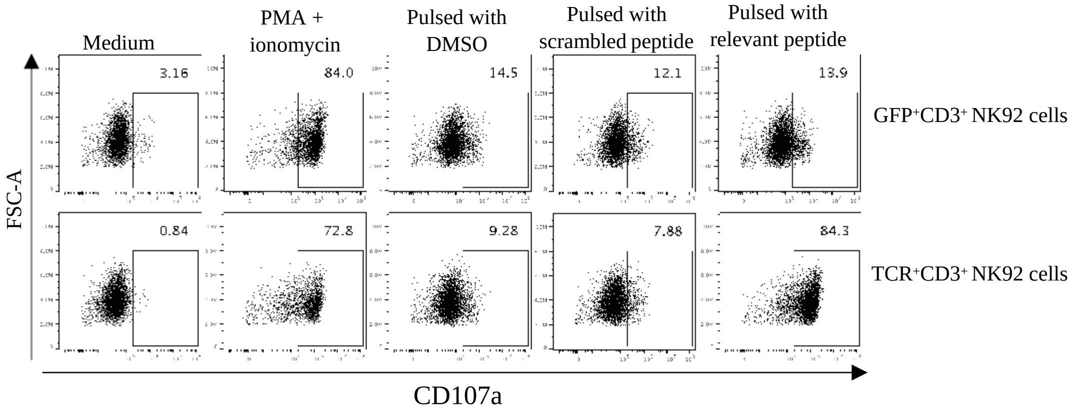

Supplementary figure S6: Flow cytometry dot plot images of CD107a positive NK92 cells after incubating with target cells.

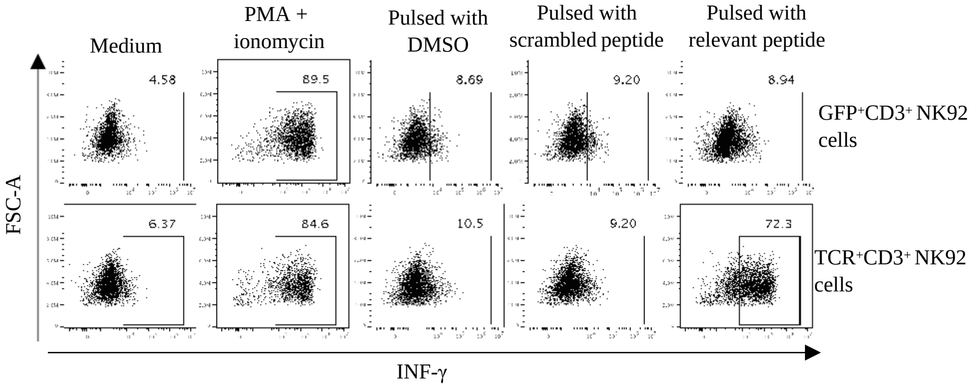

Supplementary figure S7: Flow cytometry dot plot images of IFNγ positive NK92 cells after incubating with target cells.

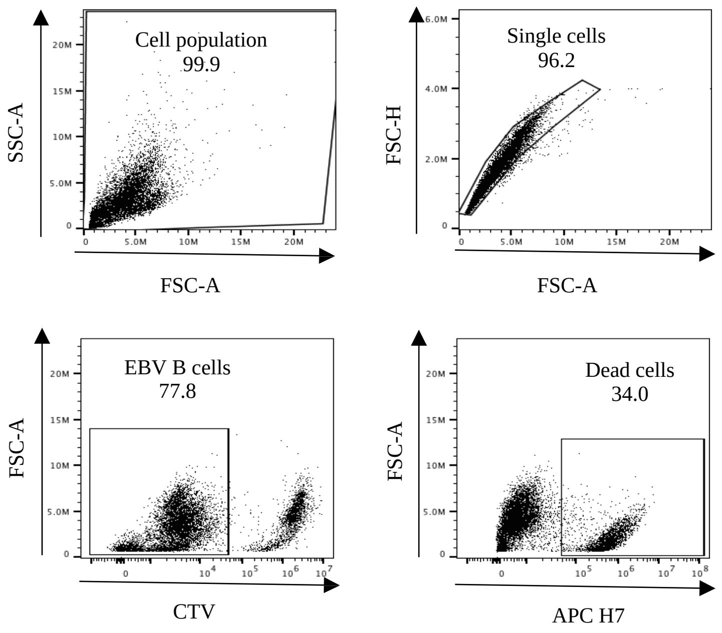

Supplementary figure S8: Gating strategy to show flow cytometry data analysis and discriminate between effector and target cells of flow cytometry-based *In Vitro* killing assay.

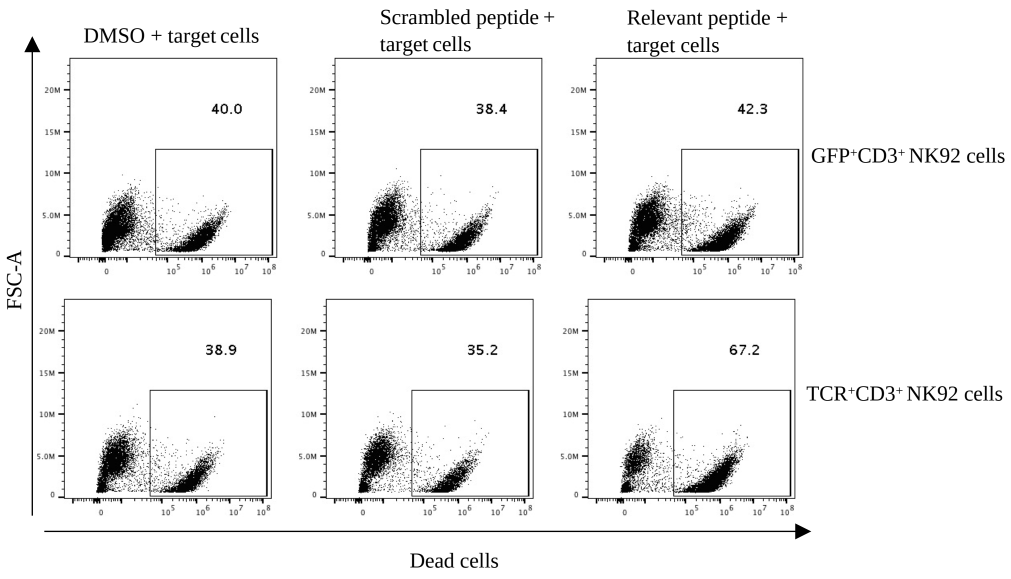

Supplementary figure S9: Flow cytometry-based *In Vitro* killing assay dot plot images. After coculturing, whole-cell populations were stained for flow cytometry determinations. Flow cytometry data were analyzed by FlowJo; dead cells were gated from CTV negative cells.

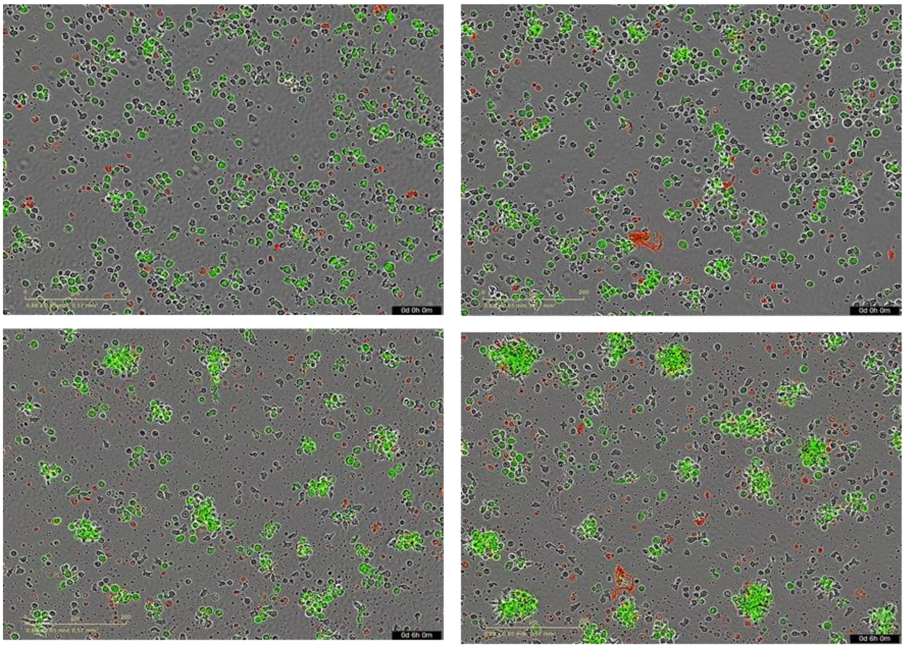

Supplementary figure S10: Live cell images of coculturing GFP$^+$CD3$^+$ NK92 cells with target cells at two different time points.

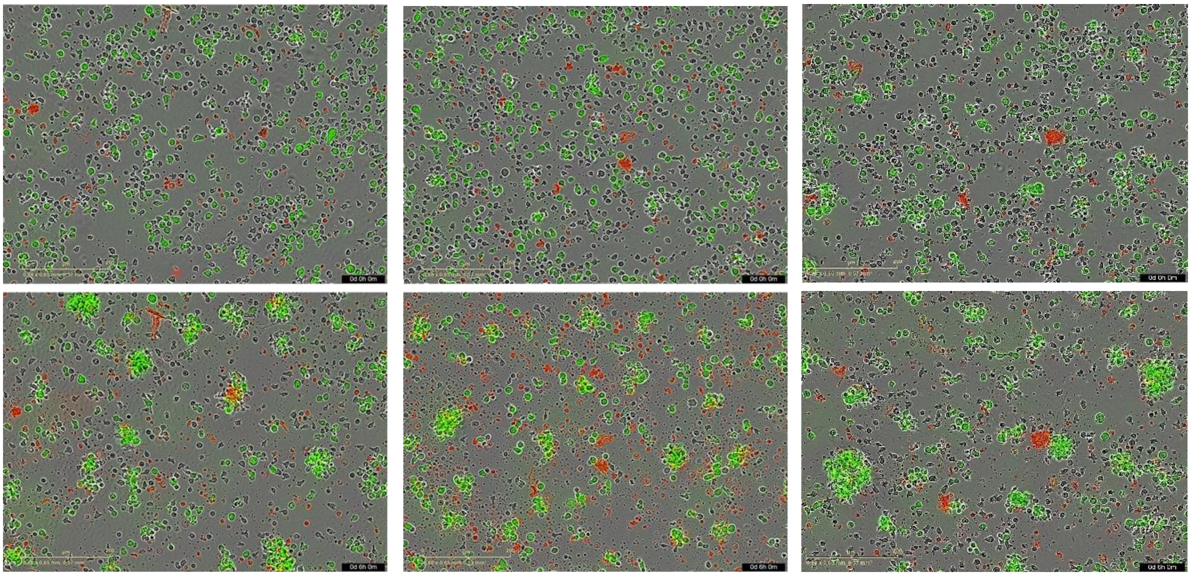

Supplementary figure S11: Live cell images of coculturing TCR$^+$CD3$^+$ NK92 cells with target cells at two different time points.

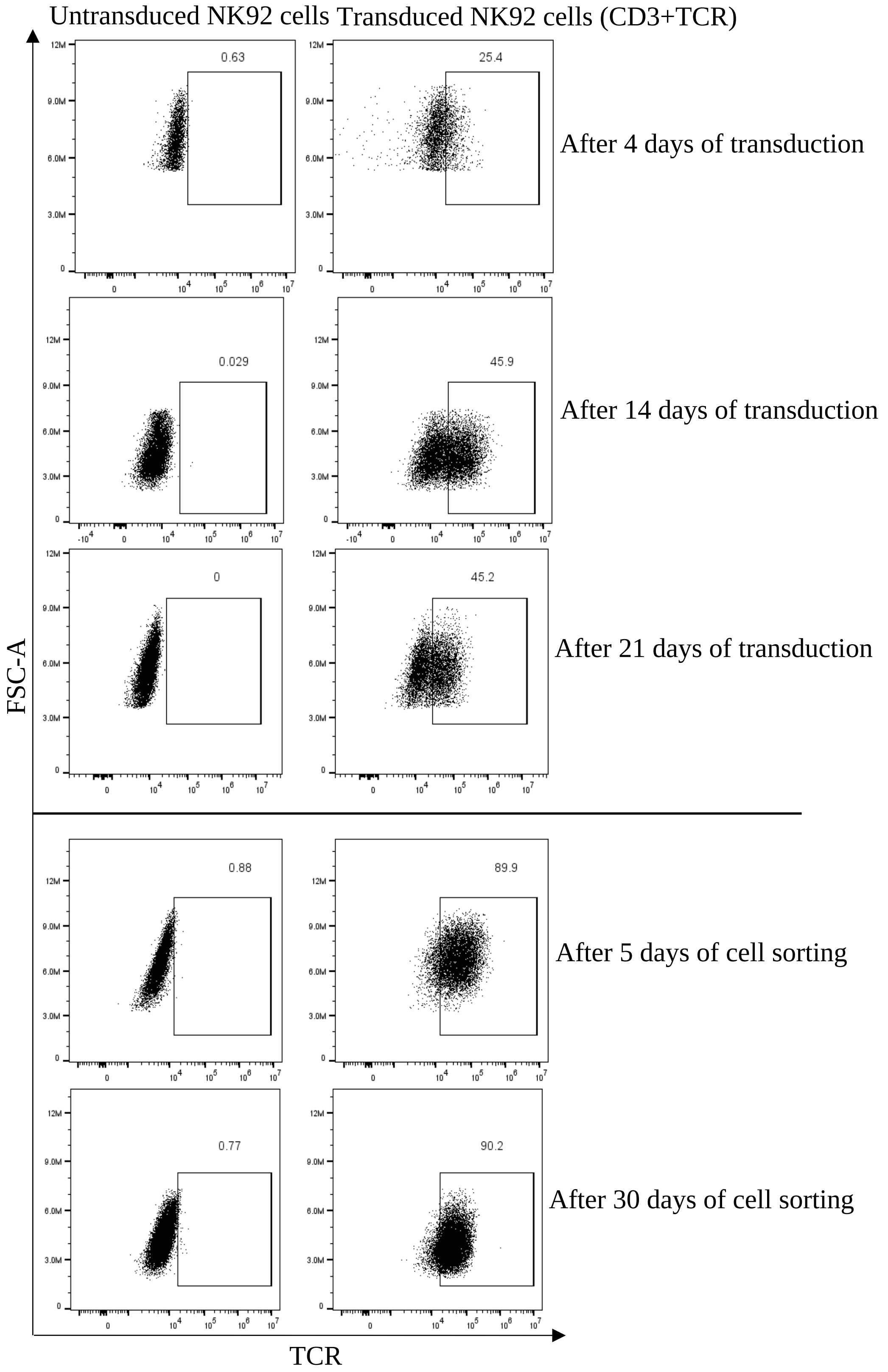

Supplementary figure S12: Flow cytometry dot plot images of TCR expression in the plasma membrane of NK92 cells in transduced and then sorted cells at different times.
